# Notes, Queries and Formulæ

**Published:** 1878-09-20

**Authors:** 


### *NOTES, QUERIES AND FORMULÆ.*

### CAPILLARY BRONCHITIS.

*Ed. Med. Record*—

DEAR SIR—In a card which I had the honor to have appear in a number of your excellent Journal, for 1878, I asked for the opinion of a competent Southern Physician as to pathology and treatment of so-called typhoid pneumonia—the capillary bronchitis of Flint's Practice. In the few remarks you had the kindness to append I noticed that you made use of the interrogation point following the title "Capillary Bronchitis," intimating, as I understood it, your doubts as to identity of the two diseases—Typhoid Pneumonia and Capillary Bronchitis. I now have the pleasure of referring you to an article by Dr. Ford, in volume 34, No. 2, for Feb. '78, St. Louis *Med. Journal,* entitled "Croupous Pneumonia;" and, also, an article by Dr. Tyndale, of New York, volume 34, No. 3, entitled "Catarrhal Pneumonia," in a series of papers on Phthisis Pulmonis. I think those gentlemen fully sustain me.

Very truly,
S. H. ANDERSON.

We make no special issue with our friend on the nice pathological point referred to in the above note. We understand Bronchitis to be an inflamation of the lining membrane of the bronchial tubes, and Typhoid Fever to be a specific, contagious, self-limited disease. When the bronchial tubes merge into capillary structure, they constitute with the connective tissue, substance of the lung itself; and thetermpneumoniashould be applied to its inflamed condition. The position of Dr. A. seems to imply that *capillary bronchitis* constitutes the disease called Typhoid Pneumonia; whereas we regard the inflamation in these cases as usually secondary, developing not unfrequently many days after the onset of the disease. In some cases we have local inflamation of other organs, as, for instance, the liver. As well might we say in such a case, that the hepatitis is the cause, rather than accompanyment of the Typhoid attack.

W.

---

The following valuable formulæ have been contributed by J. King Merritt, M. D., who has used them with great success in his practice:

No. 1.—FOR INTERMITTENT FEVER WITH CONGESTION OF LIVER.

R.—Liquid lactopeptine..............6 drachms.
Fl. ex. cinchona comp............1 drachm.
Fl. ex. taraxacum........................
Tinct. zingiber...............aa. 3 drachms.
Hydrochloric acid, diluted........1 drachm.
Spts. lavender comp.............2 drachms.
Sulphate quinine.................40 grains.

M. Dose—one teaspoonful every two or three hours.

Sig.—Quinine mixture or tonic mixture.

*Remarks.*—This mixture should be taken every two hours in the case of a quotidian attack, as soon after the subsidence of the paroxysms as the stomach will accept it, or even during the sweating stage, if the stomach is not especially irritable, and should be continued until the hour of anticipated paroxysms at the same rate, except during the night, from 10 p. m. to 4 a. m., as a general rule. Six to eight doses to be taken during the first interval, and if the attack does not recur, then continue the mixture daily for one week, at a rate diminished by one hour each day.

No. 2.—FOR INTERMITTENT FEVER WITH IRRITABLE STOMACH.

R.—Liquid lactopeptine..............6 drachms.
Fl. ex. cinchona comp............1 drachm.
Tinct. zingiber..................3 drachms.

Spts. lavender comp.............5 drachms.
Aromatic sulphuric acid ..........1 drachm.
Ess. menth. pip. or gaultheria, gtts.......10
Sulphate quinine.................40 grains.

M. Dose—one teaspoonful with water *ad libitum* every two or three hours, as in Formula No. 1, and in accordance with the type of the attack. Begin at the rate indicated—that is, if "tertian," every three hours, and then after first interval, if the paroxysm does not recur, continue mixture at a diminished rate each succeeding day, as indicated in remarks appended to Formula No. 1, to wit, by increasing the period of time between each dose of medicine an hour every day until a week has passed, when the frequency of dose will be reduced to three times a day, at which rate it should be continued until complete restoration of appetite and strength.

No. 3.—FOR MALARIAL DYSPEPSIA.

R.—Liquid lactopeptine...............dr. fl. vi.
Fl. ex. cinchona comp......................
Tinct. nux vomica............ ...aa. dr. xi.
Spts. lavender comp...................oz. ss.
Hydrochloric acid, diluted ...........dr. ss.
Syr. aromatic rhubarb ...............oz. ss.
Sulphate quinine .....................dr. ss.

M. Dose—one teaspoonful with water *ad libitum* at meals, before or after, and at bed time if required; also, use in addition, after the meals, full doses of pulverized lactopeptine with spirits lavender comp. and lime water, in case the patient should suffer from positive signs of indigestion, although the dose of Formula No. 3 has already been taken at the meal time, either immediately before or after eating, in accordance with the rule or foregoing instruction.

No. 4.—FOR CHRONIC DIARRHŒA.

R.—Liquid lactopeptine...... .......6 drachms.
Liq. opii comp. (Squibbs)........3 drachms.
Nitric acid, dilut., or aqua regia, dilut...dr. 1
Syr. aromatic rhubarb ..........2 drachms.
Pulv. nit. bismuth...................dr. ss.
Aqua camph.......................oz. ss.

M. Dose—one teaspoonful with water after each flux from bowels, and, as a rule, at bed time, even if the diarrhœa is apparently checked at that hour, and this rule should be persisted in for two or three days, or until the diarrhœal tendency has been entirely subdued.

---

CITRATE OF IRON, QUINIA, AND STRYCHNIA.

Citrate of iron and quinia (soluble)...256 grains.
Citrate of iron and styrychnia (1 per cent)...........................128 grains.
Water, warm........................ 1 fl. oz.
Simple elixir....................... 15 fl. oz.

Dissolve and mix. Citrate of iron and strychnia, of the U. S. Ph. of 1870, contains only 1 per cent of strychnia, while that of the U. S. Ph. of 1860 contained 2 per cent. One teaspoonful of the above elixir contains 2 grains of citrate of iron and quinia and 1 grain of citrate of iron and strychnia; or about $\frac{1}{100}$ grain of strychnia citrate.

---

ELIXIR OF CALISAYA.

Fluid extract of cinchona............... 3 fl. oz.

Simple elixir.........................13 fl. oz.

In this condition, this elixir should not be mixed with preparations of iron, as it would become dark-colored. To facilitate its filtration, it may advantageously be made by mixing the fluid extract with all the ingredients for the simple elixir, except the syrup, filtering through paper, and then adding the syrup.

---

GOULD'S DIARRHŒA MIXTURE.

R.—Tinct. rhei com....................1 ounce.

Tinct. opii........................½ ounce.

Tinct. camphoræ................2 drachms.

Aquæ ammoniæ.................1 drachm.

Ol. meth. pip.. ½ drachm.

Dose for an adult—one teaspoonful in hot sweetened water, repeated as often as necessary, until relief follows.

---

AILANTHUS GLANDULOSA IN DYSENTERY.

Dr. Cobert, medical chief of the British Navy in China, extols the bark of the root of this tree as superior to ipecacuanha, or any other drug, in the treatment of dysentery. It is intensely bitter, like quinia, and produces vomiting when freely used. Dr. Cobert found the dried bark of the root as good as the recent. The Chinese physicians who employ it give a cup of the strong infusion twice a day. The tree grows luxuriantly in all parts of the United States, having been introduced for the purpose of shade. It is a very rapid grower, and propagates itself abundantly by shoots from the root, being almost a nuisance in this respect. The tree is quite common in California, and is known as the ailanthus, or pride of China. Some further account of its remedial application may be found in the *New Remedies.*

### SULPHATE OF MANGANESE.

Sulphate of manganese is recommended as a substitute for calomel. It tastes somewhat like epsom salts, and is given in doses of ten to twenty grains dissolved in water, adding a little citrate of magnesia to cause effervescence. It produces dark, bilious dejections. Dr. Goolden, in the London *Lancet,* says he has employed this medicine in both private and hospital practice for thirty-four years. Strange that so little has been said or known heretofore in regard to this drug.

### THE HYPODERMIC USE OF CHLORAL IN CHOLERA.

In a late paper by Surgeon A. R. Hall, cases are reported of the successful use of chloral, hypodermically, in cholera cases, even in apparently hopeless conditions of collapse. The solution used was one part of chloral to ten parts of water; from nine to eighteen grains of the chloral were injected, and repeated according to the urgency of the case. In cholera morbus, we suppose the same good results would follow its use.

### ELIXIR OF CALISAYA AND BISMUTH.

Ammonio-citrate of bismuth..........256 grains.
Fluid extract of cinchona............ 3 fl. oz.
Simple elixir........................ 13 fl. oz.

Dissolve the ammonio-citrate of bismuth in the simple elixir, adding, if necessary, a few drops of ammonia to facilitate solution; then add the fluid extract and filter.

### ELIXIR OF VALERIANATE OF AMMONIUM.

Ammonium valerianate..............256 grains.
Simple elixir...................... 16 fl. oz.

Dissolve and mix. Each teaspoonful contains 2 grains of the salt.

It is customary to color this elixir; for this purpose a sufficient quantity of tincture of cochineal may be used; a more preferable coloring matter, however, is furnished by the berries of *Vaccinium Myrtillus*, bilberry or whortleberry, which is highly recommended for this purpose by one of our correspondents.

### ONYCHIA MALIGNA.

The free application of the subnitrate of bismuth is recommended as the best remedy for Onichia Maligna, that troublesome and painful affection of the finger nails, known as *run-rounds.* It is properly paronychia, and is one of the forms of *whitlow* or *felon.*

### ELIXIR OF QUINIA AND IRON.

Citrate of iron and quinia (soluble)...256 grains.
Water.................................. 1 fl. oz.
Simple elixir.......................... 15 fl. oz.

Dissolve and mix. One teaspoonful contains 2 grains of citrate of iron and quinia.

### COUGH SYRUP.

Dr. Kessler says, in "A. M. Journal": "We are called frequently in our everyday practice to make up a cough medicine for some of our patients. I have used all the expectorant formulas I have seen, besides a great many of my own mixtures, but so far I have obtained the most flattering results from the following:

R.—Pix liquida..................... 20 drops.
Spts. nitr. dulc................. 1 drachm.
Syr. simpl....................... 2 ounces.

M. S. Teaspoonful night and morning. Very few doses will suffice in most cases." — *Drug Circ. and Chem. Gaz.*

